# An aphid effector promotes barley susceptibility through suppression of defence gene expression

**DOI:** 10.1093/jxb/eraa043

**Published:** 2020-01-28

**Authors:** Carmen Escudero-Martinez, Patricia A Rodriguez, Shan Liu, Pablo A Santos, Jennifer Stephens, Jorunn I B Bos

**Affiliations:** 1 Cell and Molecular Sciences, The James Hutton Institute, Dundee, UK; 2 Division of Plant Sciences, School of Life Sciences, University of Dundee, Dundee, UK; 3 Helmholtz Zentrum München, Institute of Network Biology (INET), Munich, Germany; 4 University of Edinburgh, UK

**Keywords:** Aphid effector, barley, defence gene expression, host species, hormone signalling, susceptibility

## Abstract

Aphids secrete diverse repertoires of effectors into their hosts to promote the infestation process. While ‘omics’ approaches facilitated the identification and comparison of effector repertoires from a number of aphid species, the functional characterization of these proteins has been limited to dicot (model) plants. The bird cherry-oat aphid *Rhopalosiphum padi* is a pest of cereal crops, including barley. Here, we extend efforts to characterize aphid effectors with regard to their role in promoting susceptibility to the *R. padi–*barley interaction. We selected three *R. padi* effectors based on sequence similarity to previously characterized *Myzus persicae* effectors and assessed their subcellular localization, expression, and role in promoting plant susceptibility. Expression of *R. padi* effectors RpC002 and Rp1 in transgenic barley lines enhanced plant susceptibility to *R. padi* but not *M. persicae*, for which barley is a poor host. Characterization of Rp1 transgenic barley lines revealed reduced gene expression of plant hormone signalling genes relevant to plant–aphid interactions, indicating that this effector enhances susceptibility by suppressing plant defences in barley. Our data suggest that some aphid effectors specifically function when expressed in host species, and feature activities that benefit their corresponding aphid species.

## Teaser

Functional characterization of cereal pest effectors reveals virulence activities in barley, such as promoting barley susceptibility through suppression of defence and hormone signalling genes.

## Introduction

Similar to plant pathogens, aphids form close associations with their hosts and secrete effector molecules to modulate host cell processes to their benefit. Over the past decade, a combination of genomics- and proteomics-based approaches allowed the identification of putative effectors from different aphid species, including economically important pests of both monocot and dicot crops (Cooper *et al.*, 2011; Nicholson *et al.*, 2012; Atamian *et al.*, 2013; Rao *et al.*, 2013; Vandermoten *et al.*, 2014; Thorpe *et al*., 2016, 2018; Zhang *et al.*, 2017). Comparative analyses of aphid effector repertoires across species has revealed core and diverse sets, and provided insight into effector diversity and evidence for a shared transcriptional control mechanism driving their expression (Thorpe *et al*., 2016, 2018; Boulain *et al.*, 2018). Moreover, functional characterization of aphid effectors increased our understanding of how these proteins may function to enhance plant susceptibility during infestation (as reviewed by Yates and Michel, 2018 and Nalam *et al.*, 2019), and pointed to host-specific effector activities (Pitino and Hogenhout, 2013; Elzinga *et al.*, 2014; Rodriguez *et al.*, 2017).

The C002 salivary protein was first described as an effector in *Acyrthosiphon pisum*, and promotes host susceptibility to aphids (Mutti *et al.*, 2008). However, while expression of MpC002 (*Myzus persicae* C002) in host species Arabidopsis and *Nicotiana benthamiana* enhances susceptibility to *M. persicae*, expression of ApC002 from *A. pisum* in these same plant species has no visible impact on the host interaction with *M. persicae* (Pitino and Hogenhout, 2013). The difference in effector activity was attributed to a motif sequence (NDQGEE) in the N-terminal region of MpC002, which is lacking in ApC002 (Pitino and Hogenhout, 2013). In addition, several effectors from the broad host range pest *M. persicae* have been implicated in promoting host susceptibility, including Mp1 and Mp58 (Pitino and Hogenhout, 2013; Elzinga *et al.*, 2014; Rodriguez *et al.*, 2017). However, the underlying mechanisms by which these effectors impact susceptibility remain largely unknown. We previously described that Mp1 associates with the host trafficking protein VPS52 (vacuolar sorting associated protein 52) to promote plant susceptibility (Rodriguez *et al.*, 2017). Using different combinations of Mp1 and VPS52 variants from different plant and aphid species, respectively, we showed that the Mp1–VPS52 association is highly specific to the broad host range pest *M. persicae* and its hosts, and is probably shaped by plant–aphid co-evolution. Critically, effector–host protein interactions correlate with effector virulence activities. The Mp1 and Mp58 effectors and their putative orthologues are genetically linked across the genomes of at least five different aphid species (Thorpe *et al.*, 2018). Although a functional link between Mp1 and Mp58 remains to be elucidated, Mp58 was previously implicated in plant–aphid interactions. For example, Elzinga *et al.* (2014) observed a decrease in *M. persicae* performance when Mp58 was ectopically expressed in *Nicotiana tabacum* or transgenic Arabidopsis lines. In contrast, the Mp58-like effector from *Macrosiphum euphorbiae* (also called Me10) enhances tomato and *N. benthamiana* susceptibility to *M. euphorbiae* and *M. persicae* (Atamian *et al.*, 2013). Me10 was recently reported to interact with tomato 14-3-3 isoform 7 (TFT7), which contributes to defence against aphids (Chaudhary *et al.*, 2019).

*Rhopalosiphum padi* is an aphid species with a narrow host range, which includes grass species, such as barley, oats, and wheat (Blackman and Eastop, 2000). This aphid is an important pest of cereal crops that causes feeding damage and transmits some of the most destructive viruses of cereals, such as *Barley yellow dwarf virus* (BYDV). Whilst *R. padi* is highly specialized on cereals, other species, such as *M. persicae*, feature an exceptionally broad host range that includes >4000 different plant species (Blackman and Eastop, 2000), including the model plants Arabidopsis and *N. benthamiana*. Despite its broad host range, *M. persicae* is not a pest of barley and performs poorly on this plant species (Escudero-Martinez *et al.*, 2017). Recently, *M. persicae* and *R. padi* effector repertoires were identified and compared, allowing the extension of effector characterization studies to cereal pests (Thorpe *et al*., 2016, 2018). Functional characterization of aphid effectors across different plant species, including cereals, is important to gain insight into how sequence variation among effector repertoires impacts host susceptibility.

Here, we characterized three *R. padi* effectors with regards to their subcellular localization, gene expression, and contribution to susceptibility in host barley and non-host *N. benthamiana* plants. We found that expression of the *R. padi* effectors Rp1 and RpC002 in transgenic barley lines enhances plant susceptibility to *R. padi* (host interaction) but not to *M. persicae* (poor host interaction), highlighting the importance of these effectors for barley colonization in an aphid species-specific manner. Further characterization of Rp1 transgenic barley lines revealed reduced expression of several markers of plant hormone signalling pathways relevant to plant–aphid interactions, suggesting that this effector may enhance susceptibility by suppressing plant defences.

## Materials and methods

### Aphid cultures

Aphids used for the experiments were raised inside cages under controlled conditions in growth chambers (18 °C, 16 h light). *Rhopalosiphum padi* was raised on *Hordeum vulgare* L. cv. Optic, and *M. persicae* (genotype O) was reared on *Brassica napus*. The aphid species used were kindly provided by Alison Karley (JHI, UK) and Gaynor Malloch (JHI, UK).

### Identification of putative effector orthologues and plasmid construction

Effector annotation and identification of orthologues was performed as described by Thorpe *et al.* (2016). Similarity searches were performed by reciprocal best BLAST hit analysis between *R. padi* and *M. persicae* transcriptomes with the minimum thresholds of 70% identity and 50% query coverage. Pair-wise sequence analysis was performed in Jalview 2.10.4 (Waterhouse *et al.*, 2009) with T-coffee and default parameters. Signal peptide sequences were predicted with SignalP 4.1 (Petersen *et al.*, 2011). Coding sequences were amplified from *R. padi* and *M. persicae* cDNAs, without the region coding for the signal peptide, and verified by sequencing (for primers see Supplementary Table S1 at *JXB* online). The resulting amplicons were cloned by Gateway technology into pDONR201, pDONR207, or pENTR_D-TOPO (Gateway^®^, Invitrogen). Sequence-verified inserts were cloned into different destination vectors by LR reaction. Destination vectors pB7WGF2 [35S promoter, N-terminal green fluorescent protein (GFP)] and pB7WG2 (35S promoter, no tag) (Karimi *et al.*, 2002) were used for transient overexpression in *N. benthamiana*, and pBRACT214m (maize ubiquitin promoter, no tag), kindly provided by Abdellah Barakate (JHI) (Colas *et al.*, 2019), was used for generating transgenic barley lines.

### Effector gene expression in aphids exposed to host-, non-host, and poor-host plants, and artificial diet

The experimental set-up for determining aphid effector gene expression in aphids exposed to the different feeding environments is explained in detail in Thorpe *et al.* (2018). Briefly, aphids were exposed to an artificial diet, host, poor- host, or non-host plant for 3 h and 24 h, and collected for RNA sample preparation; their transcriptome was sequenced by RNA sequencing (RNAseq). More specifically, *R. padi* was exposed to barley (host) and Arabidopsis (non-host), and *M. persicae* was exposed to Arabidopsis (host) and barley (poor host). Both aphids were also exposed to artificial diet for 3 h and 24 h. A total of five independent replicates were used for this experiment, and differential expression analyses were performed as described (Thorpe *et al.*, 2018). For each selected effector (Rp1, RpC002, Rp58, Mp1, MpC002, and Mp58), we performed BLAST searches against the RNAseq data sets described in Thorpe *et al.* (2018) to identify their corresponding gene models. Transcripts were normalized by the fragments per kilobase of exon per million reads mapped (TMM-FPKM) method, which normalized the gene counts to the gene length and the library size (Conesa *et al.*, 2016).

### Effector localization

Effectors were cloned into pB7WGF2 and the constructs were transformed into *Agrobacterium tumefaciens* strain GV3101. *Agrobacterium* cells were harvested by centrifugation (8 min, 6000 rpm) and resuspended in infiltration buffer (acetosyringone 125 μM and MgCl_2_ 10 mM) to an optical density of OD_600_=0.1. *Agrobacterium* carrying the GFP–effector constructs were then infiltrated in *N. benthamiana* leaves. RpC002 and MpC002 were expressed in *N. benthamiana* transgenic line CB173 expressing the plasma membrane marker mOrange-LTi6b (Wang *et al.*, 2017). RpC002 and MpC002 were also co-expressed with the p19 silencing suppressor (OD_600_=0.1) to improve expression and thereby detection under the confocal microscope. All other effector pairs were infiltrated without p19. Fluorescence was observed 3 d after infiltration with a Zeiss LSM710 confocal microscope (Jena, Germany) using water dipping lenses. GFP was imaged using 488 nm excitation, and emissions were collected between 500 nm and 530 nm. The excitation wavelength for mOrange was 561 nm, with emission collected between 600 nm and 630nm. The experiment was repeated three times, and the resulting images were processed using ImageJ (Schneider *et al.*, 2012).

### Western blotting to detect GFP fusion proteins

Effectors were cloned into the pB7WGF2 vector and constructs were transformed into *A. tumefaciens* strain GV3101. *Agrobacterium* cells were treated as above and infiltrated in *N. benthamiana* leaves to an optical density of OD_600_=0.3. After 4 d, samples were harvested, and proteins were extracted with GTEN buffer (10% glycerol, 25 mM Tris pH 7.5, 1 mM EDTA, 150 mM NaCl, 0.1% NP-40, 10 mM DTT, and 1× protease inhibitor cocktail, Sigma). Western blots were incubated overnight with GFP antibody (Santa Cruz Biotechnology Inc., USA), for 1 h with anti-rabbit–horseradish peroxidase (HRP; Santa Cruz Biotechnology Inc.).

### Generation of transgenic barley lines expressing *R. padi* effectors

Each of the effectors was cloned into the destination vector pBRACT214m containing the ubiquitin promoter from maize for constitutive expression in all plant organs, and a hygromycin marker gene for selection of transgenic lines. Constructs were transformed into the *Agrobacterium* AGL1 strain, supplied with pSOUP, and delivered to the Functional Genomics Facility (FUNGEN) at the James Hutton Institute for *Agrobacterium*-mediated barley embryo transformation of the cultivar Golden Promise. After ~4 months, we obtained different barley lines regenerated from independent calli. The T_0_ generation was tested for the expression of effector genes by PCR on cDNA from the regenerated plants. RNA was extracted from T_0_ independent lines using the RNeasy Plant Mini Kit (Qiagen). RNA plant samples were DNase treated with Ambion^®^ TURBO DNA-free™. SuperScript^®^ III Reverse Transcriptase (Invitrogen) and random primers were used to prepare cDNA. The majority of these plants were positive in PCR tests using effector gene-specific primers (Supplementary Table S1). T_1_ seeds were germinated on selective media (AgarGel™ containing 100 μg ml^–1^ hygromycin) to select for transformants. Lines showing a 1:3 segregation, representing a single insertion (75% survival rate on selective media), were selected for further analyses. The Universal Probe Library (UPL-Roche Diagnostics^©^) was used to quantify effector gene expression in T_1_ barley transgenic lines ectopically expressing *R. padi* effectors. Barley cv. Golden Promise, the background genotype of the transgenic lines, was used as control. RNA from six different barley lines per construct was reverse transcribed into cDNA. Probes and primers (Supplementary Table S1) designed with the UPL System Assay Design (Roche) were tested for at least 95–105% efficiency. Internal controls were actin-2 (MLOC_78511.2) and pentatricopeptide (AK373147/MLOC_80089.1) as described previously (Escudero-Martinez *et al.*, 2017). Three technical replicates were included for each sample. Relative expression was calculated with the ΔCt method with consideration of primer efficiency. One of each of the transgenic effector lines was used as a reference line to calculate the fold change in additional lines.

Lines positive for effector expression were then bulked into T_2_ and screened for homozygosity based on complete resistance to hygromycin. Three independent homozygous lines per effector were used to perform the aphid performance assays with *R. padi* and *M. persicae*.

### *M. persicae* performance assays on *N. benthamiana*

Effectors were transiently expressed using vector pB7WG2 in *N. benthamiana* as explained above. The empty vector pB7WG2 was used as a control. Twelve infiltration sites were used per construct per biological replicate (*n*=12 per biological replicate). One day after infiltration, the abaxial side of the infiltration sites was exposed to two *M. persicae* adults enclosed in a clip cage. The following day, adult aphids were removed leaving three first instar nymphs on the underside of the leaves in a clip cage. Seven days later, *N. benthamiana* plants were replaced by freshly infiltrated plants to ensure continued expression of effectors in the plant tissue. After 14 d, the number of nymphs per adult was counted and data were analysed using one-way ANOVA (in R-studio) and the post-hoc Fisher’s protected least significant differences (LSD) test (cut-off *P*≤0.05). Three biological replicates were performed with each replicate containing 12 infiltration sites per construct.

### Aphid performance assays on barley transgenic lines

Seven-day old transgenic barley plants expressing *R. padi* effectors were infested with two first instar age-synchronized nymphs (*M. persicae*) or with two 2-day-old age-synchronized nymphs (*R. padi*). Barley cv. Golden Promise wild-type plants were used as the control. We used 6–8 plants per individual transgenic line for each biological replicate per aphid species (*n*=6–8), and four biological replicates were performed. The number of nymphs per adult was monitored at 11 d after infestation for *R. padi*, and after 14 d for *M. persicae*. The resulting data were analysed by one-way ANOVA (in R-studio) with post-hoc Fisher’s protected LSD test.

### Histochemical GUS staining

To assess β-glucuronidase (GUS) expression driven by the maize ubiquitin promoter in transgenic barley transformed with the pBRACT214m-GUS construct, we collected different organs (leaf, grain, spike, stem, and root) and stained these with 1 mg ml^–1^ X-gluc (5-bromo-4-chloro-3-indolyl-β-d-glucuronic acid, Thermo Scientific, USA) in X-gluc buffer (100 mM sodium phosphate buffer pH 7.0, 0.1% Triton X-100, 2 mM potassium ferricyanide, and 2 mM potassium ferrocyanide). Tissues were vacuum-infiltrated and incubated in darkness at 37 °C overnight. The next day, chlorophyll was removed with 1:3 acetic acid/ethanol. Pictures were taken under the dissecting microscope with a Zeiss camera.

### Quantitative RT-PCR to assess defence gene expression in Rp1 transgenic lines

Gene expression of different defence/hormone signalling pathways genes was analysed by quantitative real-time PCR (qRT-PCR). The transgenic barley Rp1 lines along with the control plants (cv. Golden Promise) were pre-germinated in Petri dishes covered with wet filter paper for 3 d in the dark at room temperature. Germinated seeds were placed on soil and grown under controlled conditions (8 h light, 22 °C, 70% humidity, and 125 μmol photons m^–2^ s^–1^). For basal gene expression, the first leaves of the plants (*n*=6 per genotype) were collected and flash-frozen in liquid nitrogen. In addition, barley plants (*n*=6 plants per transgenic line or wild-type control) were exposed to either empty clip cages or clip cages containing 30 mixed-age *R. padi* aphids. Leaf tissues enclosed within the clip cages were collected after 24 h and 72 h. The experiment was performed in three biological replicates (*n*=6 plants per transgenic or wild-type line per biological replicate) and samples were harvested at the same time of day: barley plants were treated and collected at 12.00 h for the 24 h time point and at 15:00 h for 72 h time point, avoiding any effects of the plant circadian cycle.

The local database Morex genes was used for retrieving the barley sequences, and the Roche UPL assay design centre for primer design (Supplementary Table S1). The primers were tested for efficiency (85–115%), and relative gene expression was calculated with the ΔΔCt method. Three technical replicates were included for each sample. Cycle threshold values were normalized with two reference genes, pentatricopeptide (AK373147/MLOC_80089) and ubiquitin (AK248472). Expression of these two reference genes was unaffected in our previous microarray experiment (Escudero-Martinez *et al.*, 2017). The Wilcoxon rank sum test (cut-off *P* ≤ 0.05) was used to assess differences in expression between plant genotypes and treatments.

## Results

### Effector sequence divergence between the aphid species *R. padi* and *M. persicae*

**Fig. 1.**
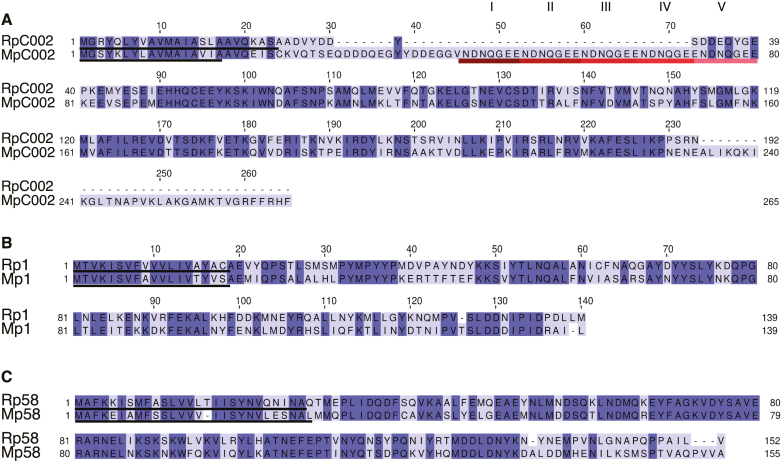
Pair-wise amino acid sequence alignments of three selected effectors from *Rhopalosiphum padi* and *Myzus persicae*. Alignments were generated using Jalview 2.10.4. The level of sequence conservation is indicated by dark (high identity) to light purple colour (low identity). Predicted signal peptide (Signal P4.1) sequences are underlined in black. (A) RpC002/MpC002 alignment. The 5× repeat motif (NDNQGEE) in MpC002 is underlined with different shades of red to pink. (B) Rp1/Mp1 alignment. (C) Rp58/Mp58 alignment. (This figure is available in colour at *JXB* online.)

We predicted putative orthologues for three previously described *M. persicae* effectors, MpC002, Mp1, and Mp58, from *R. padi* using reciprocal best blast hit analyses on available aphid transcriptome data sets and aphid genome assemblies (threshold of 70% identity and 50% query coverage) (Thorpe *et al*., 2016, 2018). To confirm the sequences of putative orthologous effector pairs, we cloned and sequenced their coding sequences. Amino acid and nucleotide sequence alignments show varying degrees of sequence divergence across the selected effector pairs (Fig. 1; Supplementary Fig. S1). RpC002 is smaller than MpC002, with 193 amino acids compared with 265, and these effectors share 52.86% sequence identity. The difference in sequence length is partly due to a lack of the NDNQGEE repeat in the N-terminal region of RpC002 (Fig. 1A; Supplementary Fig. S1). Variation in the number of NDNQGEE repeats in MpC002 was previously also detected within *M. persicae* (Thorpe *et al.*, 2016), and in this study we characterized the MpC002 version containing five repeats. MpC002 also has an extended C-terminal domain compared with RpC002 (Fig. 1A). Rp1, which is similar to *M. persicae* Mp1, is composed of 140 amino acids compared with 139 for Mp1, and these effectors share a percentage sequence identity of 56.12% (Fig. 1B; Supplementary Fig. S1). Lastly, Mp58 and Rp58 contain 152 and 155 amino acids, respectively, share 64.94% sequence identity, and are most divergent in the C-terminal region of the protein (Fig. 1C; Supplementary Fig. S1).

### Effector gene expression is consistent across different feeding/plant environments, but the range of expression varies between aphid species

**Fig. 2.**
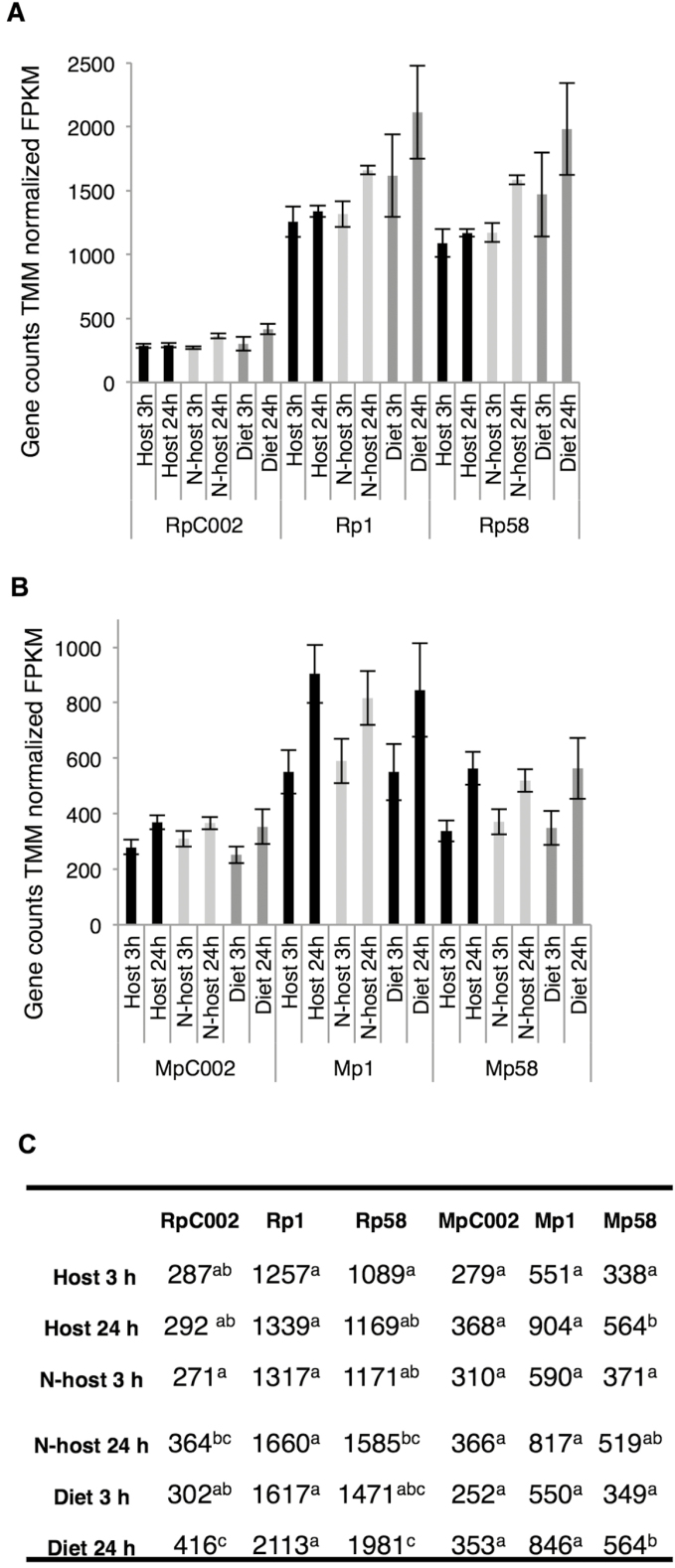
Effector gene expression in *Rhopalosiphum padi* and *Myzus persicae* upon exposure to different feeding environments. (A) Expression of *R. padi* effectors *RpC002*, *Rp1*, and *Rp58* upon aphid exposure to barley (host), Arabidopsis (non-host), or artificial diet for 3 h or 24 h. The expression of transcripts was normalized by the TMM-FPKM method (fragments per kilobase of exon per million reads mapped). Bars indicate the SE. (B) Expression of *M. persicae* effectors *MpC002*, *Mp1*, and *Mp58* upon aphid exposure to Arabidopsis (host), barley (poor-host), or artificial diet. The expression of transcripts was normalized by the TMM-FPKM method. Bars indicate the SE. (C) Table displaying expression values for each effector. Letters indicate significant differences as determined by one-way ANOVA and post-hoc protected LSD test (*P<0.05; ***P<0.01).

We were interested in assessing how gene expression of the three effector pairs was affected in *R. padi* and *M. persicae* upon exposure to different feeding/plant environments. We made use of previously generated aphid RNAseq data sets (Thorpe *et al.*, 2018) to investigate gene expression of our effectors of interest by plotting their gene counts across different treatments (exposure to diet, host, poor-host, and non-host plants) and time points (3 h and 24 h exposure). All six aphid effectors were expressed with only limited variation in expression across the different aphid treatments and time points (Fig. 2). Whilst the three selected effectors from *M. persicae* displayed more similar gene expression levels compared with one another, ranging from 280 counts for *MpC002* to 904 counts for *Mp1* (Fig. 2B, C), the three effectors from *R. padi* showed a wider range of expression. For instance, gene counts varied from 271 for *RpC002* to 2112 for *Rp1* over the various treatments and time points (Fig. 2A).

### *R. padi* effectors show similar subcellular localization to their putative *M. persicae* orthologues in *N. benthamiana*

**Fig. 3.**
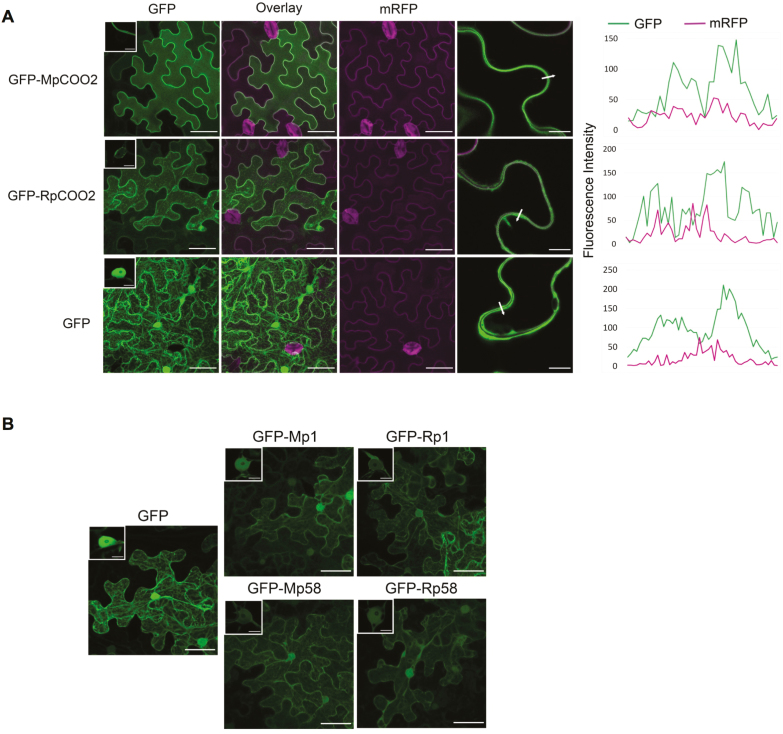
Localization of aphid effectors in *Nicotiana benthamiana.* (A) Confocal microscopy images of free GFP (empty vector, pB7WG2F), and effectors GFP–MpC002 and GFP–RpC002 (middle section) transiently overexpressed in *N. benthamiana.* Both effectors were co-expressed with a plasma membrane marker (Wang *et al.*, 2017). Merged images represent the overlay image of the GFP and mRFP (monomeric red fluorescent protein) channels. Scale bars represent 50 µm for the main images and 10 µm for the insets. The images in the last column are at higher magnification, with scale bars representing 10 µm. The arrows across the plasma membranes and apoplast of adjacent cells indicate paths used for the fluorescence intensity profiles of mRFP and GFP; the profile graphs are shown at the right of the image sets. The images were taken 3 d after agroinfiltration. The co-localization was analysed by Fiji software and the plugin RGB profiler. The images were taken 3 d after agroinfiltration. The co-localization was analysed by Fiji software and the plugin RGB profiler. (B) Confocal microscopy images of free GFP alone (pB7WG2F), and effectors GFP–Mp1/GFP–Rp1 and GFP–Mp58/Rp58. The insets show single optical sections through nuclei. Scale bars are 50 μm for the main images and 10 μm for the insets. (This figure is available in colour at *JXB* online.)

The subcellular localization of effectors can provide important information on the cellular compartment that is targeted by these proteins. We used confocal microscopy of GFP-tagged *R. padi* effectors alongside their *M. persicae* putative orthologues to compare subcellular localization *in planta*. The GFP–effector fusion proteins (N-terminal GFP tag) were transiently expressed in leaves of *N. benthamiana*, which is a host for *M. persicae*, but a non-host for *R. padi*. Western blotting showed that all GFP fusion proteins were expressed, but that two of the *R. padi* effectors, RpC002 and Rp58, showed lower protein levels than their putative *M. persicae* orthologues (Supplementary Fig. S2), with RpC002 only detected once in three biological replicates (Supplementary Fig. S2). In contrast, Rp1 from *R. padi* was detected more strongly than its putative *M. persicae* orthologue Mp1 (Supplementary Fig. S2). We detected GFP signal corresponding to the MpC002 fusion proteins by confocal microscopy at the plasma membrane of epidermal cells and, in some cases, a weak signal was present in the nucleus or the cytoplasm (Fig. 3A). In contrast, RpC002 was predominantly visible in the cytoplasm. It should be noted that expression of RpC002 was very low, especially compared with MpC002 (Supplementary Fig. S2), and only a few transformed cells were visible. We validated the plasma membrane localization of MpC002 effectors upon co-expression with a plasma membrane marker (Nelson *et al.*, 2007) (Fig. 3A). Both Rp1 and Rp58 were detected in the cytoplasm and nucleus, similar to their putative *M. persicae* orthologues and the free GFP control (Fig. 3B, C). Similarly, we tried to express tagged effectors in barley epidermal cells using particle bombardment, but, due to low signal, we were unable to reliably localize effectors in this system. Overall, the three selected *R. padi* effectors showed similar subcellular localization patterns in *N. benthamiana* to those of their putative *M. persicae* orthologues.

### Expression of Rp58 in *N. benthamiana* reduces host susceptibility to *M. persiae*

**Fig. 4.**
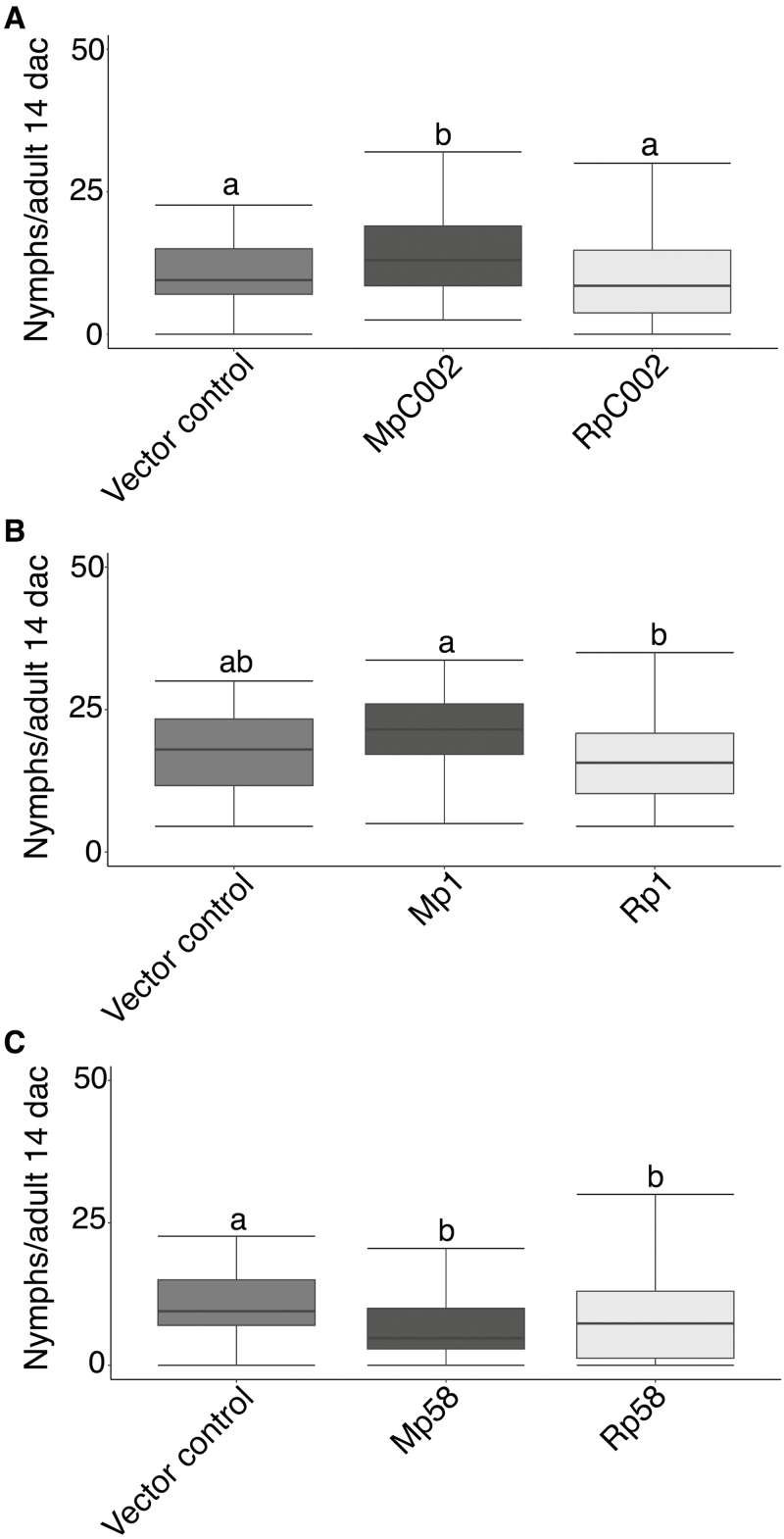
*Myzus persicae* performance on *Nicotiana benthamiana* plants expressing aphid effectors. Leaves of *N. benthamiana* were agroinfiltrated with different effector constructs (35S-promoter) and infiltration sites were challenged with three *M. persicae* nymphs, which were allowed to develop and reproduce. Nymph production per aphid was monitored over a 14 d period, with the aphids being moved to freshly infiltrated leaves every 7 d. Empty vector was used as a control. (A) Number of nymphs produced per adult on *N. benthamiana* leaves expressing the vector control, MpC002, or RpC002. (B) Number of nymphs produced per adult on *N. benthamiana* leaves expressing the vector control, Mp1, or Rp1. (C) Number of nymphs produced per adult on *N. benthamiana* leaves expressing the vector control, Mp58, or Rp58. Box plots show the average number of nymphs per adult 14 days after challenge (dac) from three independent biological replicates (number of plants per effector or control used on each replicate=12). Different letters indicate significant differences at *P*>0.05. Statistical analyses were performed using one-way ANOVA post-hoc Fisher’s protected LSD test (*P*>0.05).

To assess whether the three selected *R. padi* effectors can impact host susceptibility to *M. persicae* when expressed in an *R. padi* non-host plant species, we performed aphid performance assays on *N. benthamiana* leaves transiently expressing the different effectors under the control of a 35S promoter. In line with previous reports (Bos *et al.*, 2010; Pitino and Hogenhout, 2013), we found that ectopic expression of MpC002 significantly increased the number of *M. persicae* nymphs produced per adult by 27% (one-way ANOVA post-hoc Fisher’s protected LSD test; *P*>0.05) (Fig. 4A). In contrast, RpC002 did not alter *N. benthamiana* host susceptibility to *M. persicae*. Western blot analyses of GFP–MpC002 and GFP–RpC002 showed that RpC002 protein is detected at a much lower level than MpC002 (Supplementary Fig. S2), and therefore it is possible that the untagged MpC002 and RpC002 proteins expressed in the aphid performance assays also have different levels of abundance which affects the phenotypic observations. No significant differences in host susceptibility were noted upon expression of Mp1 from *M. persicae* and Rp1 from *R. padi* when compared with the vector control (Fig. 4B), also in line with a previous report that transient expression of Mp1 under the 35S promoter in *N. benthamiana* does not affect susceptibility (Bos *et al.*, 2010). The expression of Mp58 and Rp58 resulted in significantly lower *M. persicae* nymph production compared with the vector control, with 55% and 27% fewer nymphs being produced per adult, respectively (one-way ANOVA post-hoc Fisher’s protected LSD test; *P*>0.05) (Fig. 4C).

### Expression of RpC002 and Rp1 in transgenic barley enhances susceptibility to *R. padi*

Aphid effector characterization studies to date have focused on dicot plant species. With *R. padi* being a major pest of cereals, we aimed to extend aphid effector characterization studies to the monocot crop barley to explore the contribution of *R. padi* effectors to host susceptibility. We generated barley transgenic lines in the cultivar Golden Promise to ectopically express the three *R. padi* effectors Rp1, RpC002, and Rp58 using a modified version of the pBRACT214 vector (Colas *et al.*, 2019), containing the ubiquitin promoter from maize to allow constitutive expression in all plant organs (https://www.jic.ac.uk/research-impact/technology-platforms/genomic-services/crop-transformation/). To determine where candidate genes of interest are potentially expressed when transformed into barley using this pBRACT214m construct, we performed GUS staining of leaves, stems, spikes, grains, and roots of a barley transgenic line generated by transformation with pBRACT214m:GUS (Supplementary Fig. S3). We observed GUS expression in all organs analysed (leaf, root, grain, spike, and stem) (Supplementary Fig. S3). After barley transformation, we obtained 13 independent lines for the RpC002 effector, four lines for the Rp1 effector, and 16 lines for Rp58. In the first generation, lines with a single effector insertion were selected based on ~75% survival on hygromycin (hygromycin phosphotransferase is the pBRACT selection marker), yielding eight independent transgenic lines for RpC002, three lines for Rp1, and seven lines for Rp58. The presence of effector coding sequences (lacking the signal peptide-encoding sequence) was confirmed in the T_0_ generation by semi-quantitative RT-PCR and was verified in the T_1_ generation by qRT-PCR (Supplementary Fig. S4). We did not observe any visual differences in plant growth and development for any of the transgenic lines selected (Supplementary Fig. S4). Three homozygous T_3_ lines per effector construct were selected for aphid performance assays with *R. padi* and *M. persicae* to assess how barley host and poor-host interactions with aphids were affected. Each plant was infested with two nymphs and reproduction was assessed after 11 d for *R. padi* and after 14 d for *M. persicae.*

**Fig. 5.**
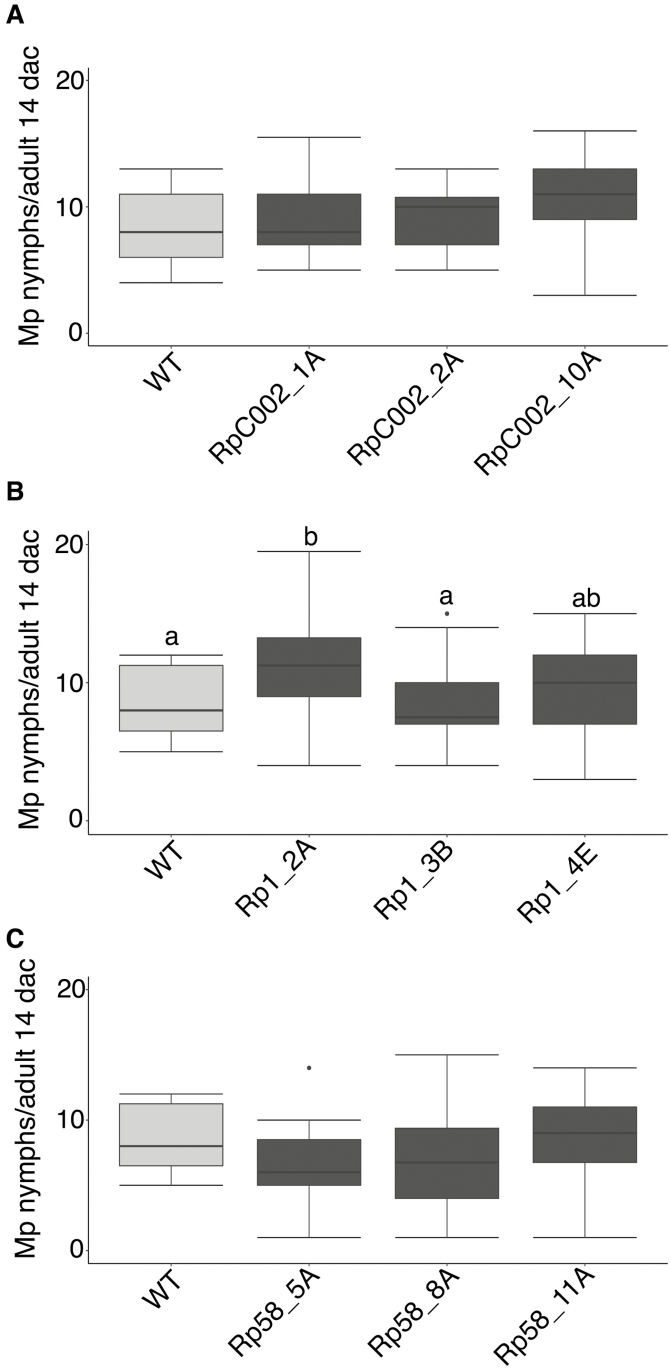
*Myzus persicae* performance on barley plants ectopically expressing different *Rhopalosiphum padi* effectors. Transgenic barley lines were challenged with aphids alongside wild-type cv. Golden Promise (WT) plants. Nymph production was monitored for 14 d. (A) Nymph production per adult on transgenic barley lines expressing effector RpC002. Three independent transgenic lines were assessed: RpC002_1A, RpC002_2A, and RpC002_10A. (B) Nymph production per adult on transgenic barley lines expressing effector Rp1. Three independent transgenic lines were assessed: Rp1_2A, Rp1_3B, and Rp1_4E. (C) Nymph production per adult on transgenic barley lines expressing effector Rp58. Three independent transgenic lines were assessed: Rp58_5A, Rp58_8A, and Rp58_11A. Box plots show the average number of nymphs per adult 14 days after challenge (dac) from at least three independent biological replicates (number of plants per effector or control used on each replicate=6–8). Different letters indicate significant differences as determined with one-way ANOVA post-hoc Fisher’s protected LSD test (*P*>0.05).

For *M. persicae*, we did not find consistent significant differences in aphid performance on the barley transgenic lines expressing the *R. padi* effectors compared with the wild-type control (Fig. 5). For one of the Rp1 lines, Rp1_2A, however, we noted increased nymph production (Fig. 5B). Line Rp1_2A was also the highest Rp1-expressing line we identified (Supplementary Fig. S4), and additional lines with similar expression levels would need to be identified to rule out the possibility that the insertion site in this line causes the enhanced susceptibility phenotype.

**Fig. 6.**
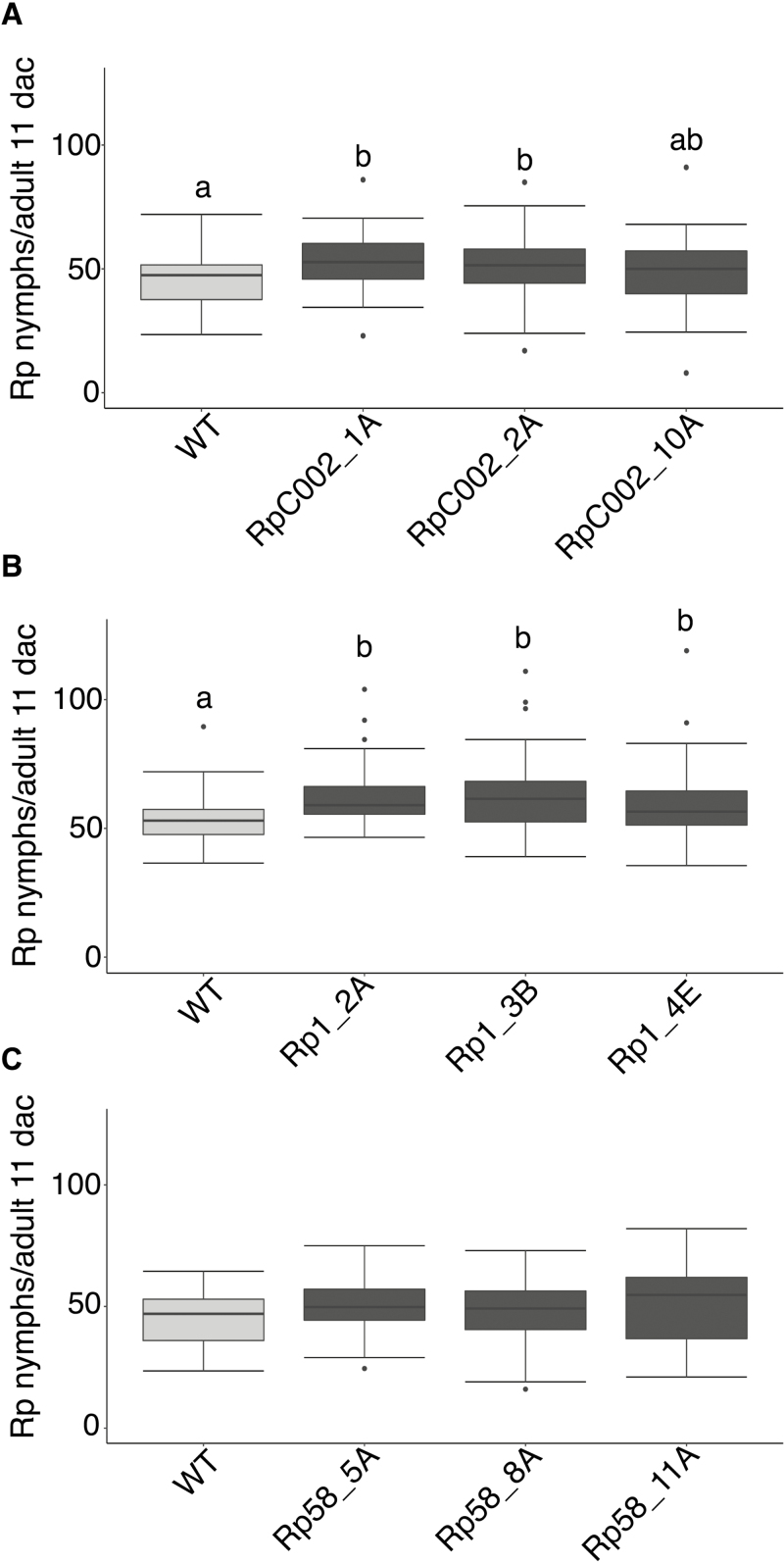
*Rhopalosiphum padi* performance on barley plants ectopically expressing different *R. padi* effectors. Transgenic barley lines were challenged with aphids alongside wild-type cv. Golden Promise (WT) plants. Nymph production was monitored for 11 d. (A) Nymph production per adult on transgenic barley lines expressing effector RpC002. Three independent transgenic lines were assessed: RpC002_1A, RpC002_2A, and RpC002_10A. (B) Nymph production per adult on transgenic barley lines expressing effector Rp1. Three independent transgenic lines were assessed: Rp1_2A, Rp1_3B, and Rp1_4E. (C) Nymph production per adult on transgenic barley lines expressing effector Rp58. Three independent transgenic lines were assessed: Rp58_5A, Rp58_8A, and Rp58_11A. Box plots show the average number of nymphs per adult 11 days after challenge (dac) from at least three independent biological replicates (number of plants per effector or control used on each replicate=5–10). Different letters indicate significant differences as determined with one-way ANOVA post-hoc Fisher’s protected LSD test (*P*>0.05).

Ectopic expression of both RpC002 and Rp1 in transgenic barley lines enhanced susceptibility to *R. padi* (Fig. 6A, B). Specifically, two out of three independent RpC002 barley lines, RpC002_1A and RpC002_2A, showed 16% and 12% increased nymph production compared with the wild-type control, respectively (one way ANOVA post-hoc Fisher’s protected LSD test; *P*>0.05) (Fig. 6A). The transgenic line with the strongest susceptibility phenotype (RpC002_1A) also showed the highest *RpC002* expression level (Fig. 6A; Supplementary Fig. S4). In addition, all three independent Rp1 barley lines showed enhanced susceptibility to *R. padi* with an increased nymph production of 11–22% across lines compared with the wild-type control (one-way ANOVA post-hoc Fisher’s protected LSD test; *P*>0.05) (Fig. 6B). Also, the level of effector gene expression seems to be correlated with the impact on host susceptibility to aphids, with the lines showing the most pronounced susceptibility phenotype towards *R. padi* (Rp1_2A and Rp1_3B) also showing the higher *Rp1* transcript levels (Fig. 6B; Supplementary Fig. S4).

No differences were observed for the Rp58 transgenic lines, which showed susceptibility levels similar to the wild-type control.

### Rp1 suppresses defence signalling in transgenic barley lines

To gain further insight into how Rp1 may enhance barley host susceptibility to aphids, we investigated the basal and induced expression levels of defence-related genes in the three independent transgenic lines we generated (lines 2A, 3B, and 4E), compared with the wild-type cultivar Golden Promise. Based on our previous work (Escudero-Martinez *et al.*, 2017), we selected barley genes strongly induced upon *R. padi* infestation: *beta-thionin* (AK252675), *SAG12*-like (MLOC_74627.1), a *jasmonate ZIM domain gene 3* (*JAZ3*, MLOC_9995), *lipoxygenase 2* (*LOX2*, MLOC_AK357253), and the *jasmonate-induced gene* (*JI*, MLOC_15761). We further expanded our selected genes set based on markers of different hormone signalling pathways, with focus on the jasmonate (JA) pathway, which is strongly activated upon aphid infestation (Escudero-Martinez *et al.*, 2017): *lipoxygenase 5* (*LOX5*, MLOC_71948), the *WRKY transcription factor 50* (*WRKY50*, MLOC_66204), the *allene cyclase oxidase* (*AOC*, MLOC_68361), and *jasmonate-induced gene 2* (*JI2*, MLOC_56924) markers for jasmonate; but also the salicylic acid (SA) marker *non-expressor of pathogenesis-related 1-like* (*NPR1*, AM050559.1), *the ethylene-response factor 1* (*EFR1*, MLOC_38561), and *abscisic acid-inducible late embryogenesis abundant 1* (*A1*, MLOC_72442). We analysed basal gene expression levels as well as expression levels upon 24 h and 72 h exposure to clip cages with or without aphids. It should be noted that the use of clip cages, even when empty, triggers changes in gene expression due to mechanical damage, and that all selected genes were induced by aphid challenge in the transgenic Rp1 lines and wild-type control (Supplementary Figs S5, S6).

**Fig. 7.**
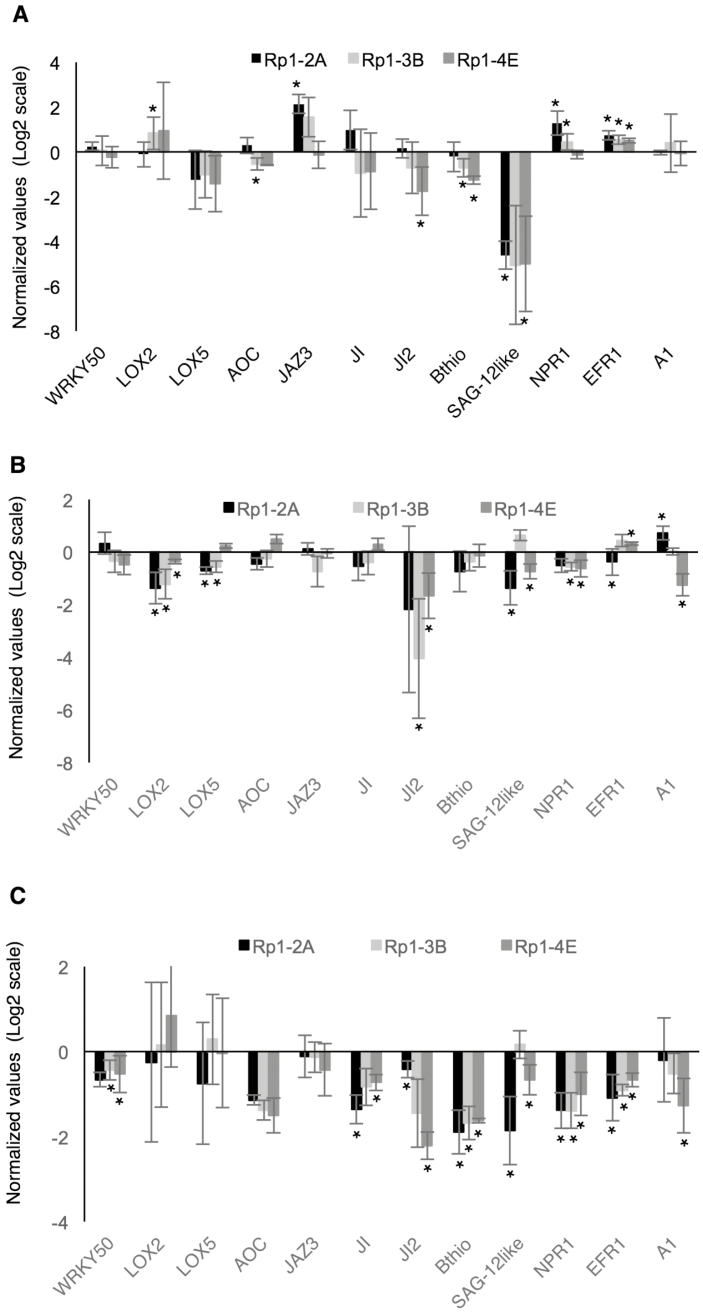
Basal and aphid-induced defence gene expression in barley Rp1 lines. Relative gene expression of defence-related/hormone signalling genes was measured by qRT-PCR in control barley plants (cv. Golden Promise) and three independent barley lines expressing the *R. padi* effector Rp1. (A) Log-fold changes of barley basal gene expression (no aphids, no clip cage) in three transgenic Rp1 barley lines relative to control lines (WT=0). (B) Log-fold changes of barley gene expression upon 24 h exposure to clip cages with *R. padi* aphids in three transgenic Rp1 barley lines relative to control plants (WT=0). (C) Log-fold changes of barley gene expression upon 72 h exposure to clip cages with *R. padi* aphids in three transgenic Rp1 barley lines relative to control lines (WT=0). All gene expression analyses were based on three independent biological replicates, and graphs represent mean expression normalized to the reference genes *pentatricopeptide* (AK373147/MLOC_80089) and *ubiquitin* (AK248472), and relative to the control lines. Genes are represented in the graphs are: *WRKY transcription factor 50* (*WRKY50*, MLOC_66204), *lipoxygenase 2* (*LOX2*, MLOC_AK357253), *lipoxygenase 5* (*LOX5*, MLOC_71948), *allene cyclase oxidase* (*AOC*, MLOC_68361), *jasmonate ZIM domain gene 3* (*JAZ3*, MLOC_9995), *jasmonate-induced gene* (*JI*, MLOC_15761), *jasmonate-induced gene 2* (*JI2*, MLOC_56924), *beta-thionin* (AK252675), *SAG12-like* (MLOC_74627.1), *non-expressor of pathogenesis-related 1-like* (*NPR1*, AM050559.1), the *ethylene-response factor 1* (*EFR1*, MLOC_38561), and *abscisic acid-inducible late embryogenesis abundant 1* (*A1*, MLOC_72442). Black bars represent gene expression levels in Rp1-2A lines, light grey bars represent gene expression levels in Rp1-3B lines, and dark grey bars represent gene expression levels in Rp1-4E lines. Asterisks indicate significant differences between control plants (WT) and Rp1 transgenic lines (Wilcoxon rank sum test, *P*≤0.05).

First, we compared basal gene expression levels across plant lines not infested with aphids and without being exposed to a clip cage (Fig. 7A). We found that expression of a gene encoding a SAG-12 like cysteine protease (MLOC_74627.1) was most strongly reduced to basal levels in Rp1 lines compared with the wild-type control, but differences were only significant for lines Rp1-2A and Rp1-4E, possibly due to sample variation for line Rp1-3B (Fig. 7A). *SAG12-like* expression was also reduced in the transgenic lines upon exposure to either empty clip cages (Supplementary Fig. S5A, B) or clip cages containing aphids (Fig. 7B, C), compared with wild-type plants, but not consistently to statistically significant levels. The *EFR1* basal expression was slightly but significantly higher in Rp1 transgenic lines compared with the wild-type control (Fig. 7A).

In addition, four genes (*WRKY50*, *AOC*, *beta-thionin*, and *NPR1*) were significantly less expressed across all transgenic lines compared with the wild-type control when leaves were exposed for 24 h to empty clip cages (Supplementary Fig. S5A, B). *LOX2*, *JI*, and *JI2* showed a trend towards reduced expression in transgenic lines, but differences were not consistently significant across all lines (Supplementary Fig. S6A, B). In response to clip cages with aphids for 24 h, only *LOX2* showed a significant reduction in expression in all transgenic lines, whereas *JI2* reduced expression was noticeably not consistently significant (Fig. 7B). For the 72 h time point, three marker genes (*beta-thionin*, *NPR1*, and *EFR1*) showed a significant reduction in expression in all lines compared with wild-type plants when exposed to clip cages containing aphids, and similar trends were observed for *WRKY50*, *JI*, *JI2*, and *SAG12-like* (Fig. 7C). Overall, we observed a reduction of several marker genes of defence/hormone signalling pathways relevant to plant–aphid interactions in the Rp1 transgenic barley lines, which may translate into their enhanced susceptibility to aphids.

## Discussion

Aphids are damaging pests on cereals, including barley. Aphid effector characterization efforts to date have focused on dicot plant species including Arabidopsis, tomato, and *N. benthamiana* (Mutti *et al.*, 2008; Bos *et al.*, 2010; Pitino *et al.*, 2011; Atamian *et al.*, 2013; Pitino and Hogenhout, 2013; Elzinga *et al.*, 2014; Rodriguez *et al*., 2014, 2017; Chaudhary *et al.*, 2019), and have not yet been described for monocot crops. It is crucial to understand the mechanisms employed by aphids and other insects to infest cereals, as well as to gain insight into how aphid effector function may have diverged across different plant–aphid species interactions. Although challenging, functional characterization of aphid effectors not only in dicot (model) plants, but also in monocot crops, promises to reveal novel insight into effector function and evolution.

Effector diversity across different plant parasites might reflect the adaptation to different host plants (Schulze-Lefert and Panstruga, 2011). Amino acid alignments of the putative orthologous aphid effectors we selected showed different levels of sequence divergence which might reflect the different lifestyles of the two aphid species *R. padi* (cereal specialist) and *M. persicae* (broad host range pest). In general, the signal peptide sequences of these effectors tend to be more conserved than their C-terminal regions, indicating that divergence mainly occurred within the functional effector domains. The NDNQGEE repeat motif, which is absent in RpC002, was previously shown to be linked to virulence in *M. persicae*, since MpC002 transgenic Arabidopsis lines, but not lines expressing a deletion mutant missing the repeat motifs, showed enhanced susceptibility to aphids (Pitino and Hogenhout, 2013). We noticed that the RpC002 protein, which lacks the NDNQGEE repeats, is less expressed/stable in *N. benthamiana*, which could explain the limited impact on plant susceptibility in this species. It is noteworthy that, within *M. persicae*, different MpC002 variants have been reported with different numbers of the NDNQGEE repeat (Thorpe *et al.*, 2016). The biological significance of this repeat variation remains to be elucidated.

All selected aphid effectors were expressed regardless of whether aphids were exposed to a host, non-host plant, or artificial diet (Fig. 2). It is possible that, unlike the case for plant pathogens where effector gene expression varies across different infections stages (O’Connell *et al.*, 2012; Hacquard *et al.*, 2013; Jupe *et al.*, 2013; Cotton *et al.*, 2014), aphid effectors are constitutively expressed to ensure aphids are generally ready to infest a plant. This hypothesis is in line with other reports where no significant overall effector gene expression variation was reported when aphids were adapted to different plant environments (Lu *et al.*, 2016; Mathers *et al.*, 2017; Thorpe *et al.*, 2018). The Rp1–Mp1 and Rp58–Mp58 pair was more similarly expressed in the two aphid species relative to the RpC002–MpC002 effector pair. Interestingly, the Mp1- and Mp58-like effectors are co-located in a non-syntenic region across the genomes of five different aphid species, and their expression is tightly co-regulated with a large set of aphid genes, including many (predicted) effectors such as MpC002 (Thorpe *et al.*, 2018). Whether and how these effectors work together to enable aphid infestation remains to be explored.

MpC002 from *M. persicae*, but not RpC002 from *R. padi*, localized at the plasma membrane in *N. benthamiana*, indicating that this could be the site of activity for this effector (Fig. 3A). Both the nucleus and the plant plasma membrane play key roles in activating plant defences against plant pathogenic microbes (reviewed by Motion *et al.*, 2015; Boutrot and Zipfel, 2017). The plasma membrane is the site of many immune receptors, such as receptor-like kinases, required for pathogen recognition and initiation of an immune response (Boutrot and Zipfel, 2017). The plasma membrane localization of these aphid effectors might reflect a role in interfering with immune receptors or any other cell membrane-associated defences. It should be noted that effector localization using highly expressed effectors (35S-based) may be affected by (endogenous) expression levels of their host targets. For example, only a small proportion of a highly expressed effector may bind to a low abundance endogenous host target in/at a specific subcellular compartment, with most of the effector detected by confocal microscopy remaining in an unbound state. This is the case for Mp1, which only co-localizes to vesicles in the presence of overexpressed VPS52 (interacting host protein), with endogenous levels of VPS52 being low in leaf tissues (Rodriguez *et al.*, 2017).

MpC002 and RpC002 differed not only in their protein expression level and subcellular localization in *N. benthamiana*, but also in their ability to promote susceptibility in this plant species to *M. persicae*, with only MpC002 expression resulting in an increase in aphid fecundity (Fig. 4A). Species-specific activity within the aphid C002 family was previously reported and linked the presence/absence of the NDNQGEE repeat motif (Pitino and Hogenhout, 2013). In contrast, RpC002 increases barley susceptibility to *R. padi*, indicating that the effector is functional when expressed in an appropriate host plant (Fig. 6A). Whether the NDNQEE motif is associated with reduced protein expression and/or stability in certain plant species remains to be investigated.

Both Rp58 and Mp58 similarly reduced *N. benthamiana* susceptibility to *M. persicae*, pointing to a potentially conserved function of these effectors (Fig. 4C). The reduction in susceptibility mediated by Mp58 is in line with a report by Elzinga *et al.* (2014). Potentially, the artificially high levels of Rp58/Mp58 expression lead to an exaggerated host targeting response and subsequent activation of defences. Alternatively, Rp58/Mp58 was not expressed in tissues where these effectors are usually delivered and active, or these proteins may only function in combination with additional effectors in enhancing plant susceptibility. In contrast to our observations, Atamian *et al.* (2013) reported that the putative orthologue of Rp58/Mp58 in *M. euphorbiae* (Me10) increased tomato and *N. benthamiana* susceptibility to the potato aphid. Perhaps these effectors function in a different way across plant–aphid interactions.

The lack of an impact of Rp1 and Mp1 on *N. benthamiana* susceptibility to *M. persicae* was not surprising as it was previously shown that Mp1, when expressed under the 35S promoter, does not alter plant susceptibility (Bos *et al.*, 2010; Elzinga *et al.*, 2014). However, when expressed under a phloem-specific promoter, Mp1, but not Rp1, enhances *N. benthamiana* susceptibility to *M. persicae* (Pitino and Hogenhout, 2013; Rodriguez *et al.*, 2017). Interestingly, Rp1 expression in barley, driven by a ubiquitin promoter, enhanced barley susceptibility to *R. padi* but not to the same extent as to *M. persicae* (Figs 5B, 6B), suggesting that not only Mp1, but also Rp1, promotes aphid susceptibility in a specific plant–aphid system. Barley resistance to *M. persicae* is probably phloem based (Escudero-Martinez *et al.*, 2019, Preprint), and barley transcriptional responses to this aphid species include a strong activation of a specific set of defence-related genes (Escudero-Martinez *et al.*, 2017). It is possible that effectors from the cereal specialist *R. padi* do not affect barley resistance mechanisms against *M. persicae* and as a result susceptibility remains comparable with that of wild-type plants.

The effect of Rp1 on barley susceptibility to *R. padi* is probably associated with the suppression of several defence genes we observed in transgenic lines expressing this effector (Fig. 7). *SAG12-like* encodes a cysteine protease involved in hypersenescence and has been implicated in Arabidopsis PAD4-mediated defence against aphids (Pegadaraju *et al.*, 2007). Barley genes encoding β-thionins contribute to defence against aphids (Escudero-Martinez *et al.*, 2017), as well as those coding for components of the JA signalling pathway (reviewed by (Züst and Agrawal, 2016). For example, *LOX2* overexpression in barley increased resistance towards *R. padi* and *M. persicae*, possibly by activating a group of JA-related genes. In line with this, knock down of *LOX2* in barley resulted in enhanced susceptibility to these same aphid species (Losvik *et al.*, 2017). The WRKY50 transcription factor is implicated in JA signalling, but negatively regulates JA responses while promoting SA-induced expression of PR1 (Gao *et al.*, 2011; Hussain *et al.*, 2018). Expression of WRKY50 is slightly reduced in the Rp1 lines compared with the control upon stress (e.g. leaf surface damage, interference with photosynthesis, and leaf gas exchange) caused by clip cages (24 h), as well as upon aphid infestation (72 h) (Fig. 7C; Supplementary Fig. S5). Moreover, the consistent reduction of both SA and ethylene signalling markers (*NPR1* and *EFR1*) 72 h after aphid exposure in the Rp1 transgenic, despite higher basal levels in most of the lines, suggests that defence pathways are suppressed upon expression of the Rp1 effector (Fig. 7C). Our work represents an important step towards understanding the function of aphid effectors promoting susceptibility in a monocot crop. The future identification of barley host targets of effectors such as Rp1 will help us further link the observed suppression of defence gene expression to host susceptibility and reveal the underlying mechanisms of effector-mediated susceptibility to aphids.

## Supplementary data

Supplementary data are available at *JXB* online.

Fig. S1. Pair-wise nucleotide sequence alignments of putative orthologous effectors from *Rhopalosiphum padi* and *Myzus persicae*.

Fig. S2. Western blots showing the expression of GFP and the GFP–effector fusion proteins in *Nicotiana benthamiana*.

Fig. S3. Expression of GUS (β-glucuronidase) under control of the maize ubiquitin promoter in different organs of the barley transgenic line generated using pBRACT214:GUS.

Fig. S4. Effector transcript levels in transgenic barley lines expressing *Rhopalosiphum padi* effectors and plant phenotypes.

Fig. S5. Defence-related gene expression in barley Rp1 lines after exposure to empty clip cages without *Rhopalosiphum padi*.

Fig. S6. Defence-related gene expression in barley Rp1 lines after exposure to clip cages with *Rhopalosiphum padi*.

Table S1. PCR primers used to clone the different effectors, and qRT-PCR primers and probes used to quantify effector gene expression.

eraa043_suppl_Supplementary_Material

## Author contributions

JIBB conceived and directed the project; CE-M and JIBB designed the experiments; CE-M, PAR, SL, and PAS performed the experiments; CE-M, PAR, SL, and JIBB analysed the data; JS generated the barley transgenic lines; CE-M and JIBB wrote the manuscript; all authors approved the final manuscript.
